# Provision of drug and alcohol services amidst COVID-19 pandemic: a qualitative evaluation on the experiences of service providers

**DOI:** 10.1007/s11096-023-01557-1

**Published:** 2023-03-27

**Authors:** Levi Richards-Jones, Priya Patel, Parbir Kaur Jagpal, Richard Lowrie, Karen Saunders, Sarah Burwood, Sunil Shrestha, Vibhu Paudyal

**Affiliations:** 1 School of Pharmacy, College of Medical and Dental Sciences, Institute of Clinical Sciences, University of Birmingham, Edgbaston, Birmingham, B15 2TT UK; 2 Pharmacy and Prescribing Support Unit, NHS Glasgow and Clyde, Glasgow, G76 7AT UK; 3 Public Health England, West Midlands, B2 4BH UK; 4 School of Pharmacy, Monash University Malaysia, Jalan Lagoon Selatan, 47500 Bandar Sunway, Selangor Malaysia

**Keywords:** COVID-19, Drug and alcohol services, Substance use disorders, United Kingdom

## Abstract

**Background:**

The impact of COVID-19 pandemic on the provision of drug and alcohol (D&A) services and associated outcomes have been under-researched.

**Aim:**

This study aimed to understand the experiences of service providers in relation to how drug and alcohol (D&A) services were affected during COVID-19 pandemic, including the adaptations made and lessons learnt for the future.

**Method:**

Focus groups and semi-structured interviews were conducted with participants from various D&A service organisations across the UK. Data were audio recorded, followed by transcription and thematic analysis.

**Results:**

A total of 46 participants representing various service providers were recruited between October and January 2022. The thematic analysis identified ten themes. COVID-19 required significant changes to how the treatment was provided and prioritised. Expansion of telehealth and digital services were described, which reduced service wait times and increased opportunities for peer network. However, they described missed opportunities for disease screening, and some users risked facing digital exclusion. Participants who provided opiate substitution therapy service spoke of improving service provider/user trust following the shift from daily supervised treatment consumption to weekly dispensing. At the same time, they feared fatal overdoses and non-adherence to treatment.

**Conclusion:**

This study demonstrates the multifaceted impact of the COVID-19 pandemic on UK-based D&A service provisions. The long-term impact of reduced supervision on Substance Use Disorder treatment and outcomes and any effect of virtual communications on service efficiency, patient-provider relationships and treatment retention and successes are unknown, suggesting the need for further study to assess their utility.

**Supplementary Information:**

The online version contains supplementary material available at 10.1007/s11096-023-01557-1.

## Impact statements

- Adjustments in D&A services during the COVID-19 pandemic brought challenges and opportunities to service providers.
- There is a need to research the long-term impact on patient outcomes of less frequently supervised models of substance misuse treatment
- The impact of virtual communications on service efficiency, patient-provider relationships and treatment retention are also important areas for future research.

## Introduction

The first positive cases of COVID-19 were identified in the UK on 29 January 2020, and the World Health Organization (WHO) declared the outbreak as a worldwide pandemic on 11 March 2020 [1]. From the week ending 13 March 2020 to the week ending 10 December 2021, the number of excess deaths above the five-year average in England and Wales was 131,277 [2]. Mitigation measures such as social distancing and stay-at-home orders were gradually introduced in late March 2020 [3, 4]. In addition, telehealth was expanded, replacing face-to-face appointments [5]. However, telehealth is likely less accessible to some of society’s most vulnerable, including those with substance use disorders (SUDs). Those with a SUD are susceptible to homelessness, mental illness, and poverty, hindering access to digital support and telehealth [6, 7].

SUD is suggested to worsen COVID-19 prognosis, particularly for those with a depressant use disorder, through exacerbation of acute respiratory distress syndrome (ARDS) associated with severe COVID-19 [8, 9]. For example, chronic excessive alcohol consumption can aggravate ARDS by impairing alveolar epithelium integrity and contributing to cirrhosis-induced immune dysfunction [10–13]. A recent European Monitoring Centre for Drugs and Drug Addiction report suggested that accessibility to illicit substances declined during the COVID-19 pandemic and led to price surges [14]. Many feared potential exploitations of persons experiencing SUD, such as forced burglary and sex work. Both social and health-related vulnerabilities suggest that drug and alcohol (D&A) service provisions are essential in protecting people with SUDs, particularly during major disasters.

Existing literature indicates that engagement between people with SUDs and D&A services during COVID-19 pandemic fell significantly. For example, in England, over a third (36%) reduction in users of needle exchange services was reported [15]. Risks associated with substance use are likely to have been elevated during the COVID-19 pandemic linked to factors such as lack of access to clean paraphernalia and shortages of illicit substances leading to users resorting to new or different substances of abuse and thereby increasing the likelihood of poisoning [16]. Services relevant to persons experiencing homelessness also faced the squeeze in resources contributed by the pandemic [17]. D&A services are necessary to protect the vulnerable population from risks associated with substance misuse, particularly during pandemics such as COVID-19. Learning from the experiences of service provision during the pandemic can inform more-resilient D&A provision enabling better responses to future challenges.

### Aim

This study aimed to understand the experiences of service providers in relation to how drug and alcohol (D&A) services were affected during COVID-19 pandemic, including the adaptations made and lessons learnt for the future.

### Ethics approval

Ethical approval was obtained from the University of Birmingham School of Pharmacy Safety and Ethics Subcommittee (UoB/SoP/2021–10)**.**

## Method

### Study design

The study was carried out in the UK between 21 October and 28 November 2021. Organisations and their contact details involved in the provision of D&A services were identified through researchers’ professional networks, public health organisations, NHS portals and Google search engines. Representatives from 139 organisations who had roles in the frontline provision or commissioning of services in 12 administrative regions of the UK were invited to participate through invitation email. Informed consent was obtained prior to the interviews and focus groups.

### Data collection

All focus groups and interviews were conducted remotely using either Microsoft Teams or Zoom videoconferencing software by LRJ and PP. Interviewees were not previously known to the participants. A topic guide (electronic supplemental material 1) comprising fourteen questions was designed based on the research aim, existing literature and research team expertise. The discussion focused on general experiences of providing drug and alcohol services during the covid-19 pandemic including counselling practices, difficulties encountered in the delivery of care, use of digital technology and perceived impact on quality of care, any perceived differences in rates of overdose during pandemic compared to pre-pandemic period, impact of the pandemic on multidisciplinary team working and communications, impact of the pandemic on the attitudes of staff towards patients and vice-versa, impact on the number of people seeking help during pandemic, any learnings from the pandemic and how services could be made more resilient for the future. Audio recordings were transcribed verbatim and anonymised. Focus groups and interviews were conducted until data saturation was achieved based on the number of emergent themes and following discussion with the research team. Two researchers (LRJ and PP) independently analysed the data and discussed it with the third reviewer (VP). Thematic framework method was used to analyse the data supported by NVivo 12 software.

## Results

Focus group discussions and semi-structured interviews were conducted with participants from various D&A service organisations across the UK. Focus group included one large forum consisting of 35 participants convened through Public Health England and another focus group consisting of five participants. This was followed by six semi-structured in-depth interviews, giving a total of 46 participants. Focus groups lasted approximately 70 min on average and in-depth interviews lasted 30 min. Participants represented various organisations, including alcohol and drug recovery services, community pharmacies and the voluntary sector. A total of 10 themes emerged from the data (Table 1).

**Table 1:** Ten themes identified during thematic analysis

Themes identified from focus groups/interviews
1. General Experiences of Service Provisions During the COVID-19 Pandemic
2. Protecting the Most Vulnerable
3. Changes to Interprofessional and Service Provider/User Communication
4. Patient-Provider Relationships During The Pandemic
5. Psychological Impact of COVID-19 on Service Providers and Service Users
6. Resource and Service Availability
7. Organisational Challenges
8. Preserving the Quality of Care
9. Self-Reported Trend in Substance Use During the Pandemic
10. Learning from the Pandemic

### General experiences of service provisions during the COVID-19 Pandemic

During the initial outbreak of the pandemic, immediate concerns as described by the participants related to the continuation of services and monitoring vulnerable individuals whilst following government guidelines. Due to the national lockdown, the overnight closure, and reductions in service capacity, there was an urgent need to adapt how in-person interventions were delivered. Participants spoke of service users offered reduced supervision from D&A services during the COVID-19 pandemic. For example, adolescent service users were monitored less frequently, which was attributed partially to the closure of schools and other organisations that liaise with D&A services. Community pharmacies dispensed several days of supply of adult opioid substitution therapy (OST), i.e. methadone or buprenorphine, at once, replacing daily supervised consumption.

“We reduced the supervision to probably once, twice or three times a week” (NHS Foundation Trust Regional Manager).

“Taking home a month’s worth from a pharmacy. That was reduced to two weeks” (Addiction Service Lead Pharmacist).

Adaptations also included substituting methadone with buprenorphine due to reduced monitoring capacity.

“We didn’t initiate anyone on methadone, […] only initiated using buprenorphine. Because we weren’t doing any urine testing, there was a risk of overdose” (NHS Foundation Trust Regional Manager).

However, some participants described that the pandemic brought opportunities to offer outreach services.

“We just did a lot of outreaches we even took on nursing services onto the streets” (Drug misuse and needle-exchange service employee).

### Protecting the most vulnerable

All participants highlighted the importance of initially risk assessing with a ‘traffic light system’ to distinguish the most vulnerable service users and assess individual needs to adjust how support was offered. Participants emphasised that although face-to-face contact was minimised, service users deemed to be in higher needs were offered more opportunities for face-to-face contact.

“In the beginning we tried to maintain contact with people in at least the proportion of contacts that we had prior to lock down, some actually we tried to have more contact with, if our assessment was there are particular risk issues or particular needs” (D&A Services Provision Manager).

Many providers described increased vulnerabilities amongst the most disadvantaged, including those relying on street begging, and sex workers, as the pandemic took away their livelihood. Other challenges that complicated service delivery included a reported increase in domestic abuse.

### Changes to interprofessional and service provider/user communication

All participants reported that telemedicine was significantly expanded following the outbreak of COVID-19. While many described that remote communications improved professional efficiency, some participants spoke of the drawbacks of telehealth and digital D&A services, citing staff computer shortages and an inability to conduct physical health examinations thoroughly. Moreover, some participants found telemedicine to negatively-impact service provider/user communication, crediting a loss of nonverbal cues and difficulty building relationships with new service users.

“If you’re unable to develop that relationship to its full extent because you’re not interacting with people in the same way, then it’s going to suffer.” (Youth D&A Misuse Service Manager).

In contrast, participants described that stable service users found remote consultations convenient. This was partially attributed to boredom and isolation due to restrictions on socialisation during lockdown periods. On the other hand, non-access to digital devices would make some service users disadvantaged. Some providers described maintaining face-to-face services to those without such access.

“Those that were really vulnerable were extremely vulnerable at that time and the divide between them was massive. So yeah, we just sprioritised risk. Those that were most at risk had the most face to face contacts and those that were able to sutilise the digital offer and we sort of use that for those that could.” (D&A recovery worker).

Digital appointments, particularly group sessions, offered greater ies to develop peer networks.

“Just being the importance of providing people the opportunity to connect with the right kind of support but also like minded peers so that they can, you know, recovered together. I think without peer support I mean the greatest success that we've had during the pandemic has been through our online group support where people are meeting daily and supporting each other and maintaining connections that they've.” (Recovery services manager, D&A service).

In addition, digital service provisions reduced the waiting time for appointments. In addition, particularly for counselling services and psychological therapies such as cognitive behavioural therapy (CBT), outcomes were deemed to have been improved, and recovery rates increased.

### Patient provider relationships during the pandemic

All participants who provided OST spoke of service provider/user trust improving following the suspension of supervised consumption of opioid substitution therapy. In addition, many service users were deemed to have felt empowered to take control of their treatment, adhering to their medication regimen.

“They […] showed us that they were trustworthy […] they were able to manage the bits we were worried about around their […] medications.” (Drug misuse and needle-exchange service employee).

Improved interprofessional collaboration between D&A and other services, such as homelessness service providers, was also described. Several subjects reported that accessibility of D&A services to homeless people improved during lockdowns, citing that secure accommodation provisions facilitated access to D&A services.

“We could look back and think why didn’t we do that, sooner, why did it take a pandemic, to get people housed, fed, clothed, nursing care delivered to them and a degree of stability, which allowed people to stick on scripts or even detox from that alcohol” (D&A Services Provision Manager).

### Psychological impact of COVID-19 on service providers and service users

Most participants described increased personal and work-related stress during the COVID-19 pandemic, citing unwell family members, self-isolation-related staff shortages, and frustration of service users.

“Staff were very pressured because a lot of them had to shield themselves, and patients were a bit frustrated” (Addiction Service Lead Pharmacist).

Some participants reported apprehension about non-adherence to treatment, overdoses and deaths due to reduced supervision and feeling depressed and isolated during lockdown periods.

“I went a bit wonky at the beginning of this year, and when I look back on it, I think it was depression” (Youth D&A Misuse Service Manager).

Participants also described a rise in anxiety and dread amongst service users. Some service providers saw an increase in the number of referrals for alcohol. Many individuals were found to have relapsed during the pandemic outbreak, particularly for alcohol, as isolation and social deprivation resulting from the lockdown may have encouraged substance and alcohol misuse as a coping mechanism, while others stockpiled medicines.

“People were stocking up on medications because they were worried about […] what lockdown one’s going to be like.” (D&A Misuse Service Regional Manager).

Conversely, other participants explained how school closures and restrictions on socialisation reduced peer pressure for some adolescent service users, resulting in reduced substance use.

“In a friendship group where your friends are offering it to you, where your friends are… However, in COVID, that isn’t the thing so, if you never started it outside of COVID, and then COVID happens, you’re less likely to be in that environment where you know where to get it.” (Youth D&A misuse service employee).

Some participants, however, explained that engagement with young people diminished as the majority of young people didn’t have a private, safe, and secure environment in which to participate in remote online telephone calls.

### Resource and service availability

Most participants spoke of insufficient resources citing understaffing and lack of available personal protective equipment (PPE), particularly during the first wave of the pandemic.

“It took us a month or so before we got the proper PPE in place.” (NHS Foundation Trust Regional Manager).

Several participants also explained how unfilled staff shortages led to the suspension of services, such as supervised consumption and screening, treatment, and vaccination of hepatitis infection.

“Hepatitis screening, hepatitis C treatment, hep B vaccinations, […] was put on hold because of this. Because we were not able to see people face-to-face” (NHS Foundation Trust Regional Manager).

Similarly, another participant reported: “We stopped the supervised diamorphine” (Addiction Service Lead Pharmacist).

Many blamed the unpreparedness of D&A services on service providers not making contingency plans seriously prior to the outbreak of COVID-19.

“I don’t think we ever rehearsed our contingency plans” (Addiction Service Lead Pharmacist).

Adolescent safeguarding provisions were also reported to have been affected, citing school closures reducing interprofessional dialogue between school-based and D&A services.

“It’s a bit more tricky to talk to professionals about it because it’s not just like, you know, you can talk to a teacher after a session and be like ‘how has this kid been doing in school.” (Youth D&A misuse service employee).

Participants noted that there were increased queues in pharmacies contributed by increased demand for prescription services which was deemed to have created enormous pressure on pharmacies to have the capacity to continue daily supervised consumption.

“Queues of pharmacies for everyone, and that made it really difficult if you were supposed to be collecting a script daily, submitted by yourself, in a queue for hours on end.” (D&A Service Provision Manager).

Services also had to introduce home delivery service to users on daily OST who had to self-isolate during the pandemic and one participant suggested this was complicated to set up in terms of permissions and authority to carry medication on behalf of service users. Supervised consumption regimes were adjusted to weekly or even monthly prescriptions instead of daily, and so individuals were now responsible for a larger supply of medication one participant noted;

“This created a little bit of anxiety for us as an organisation because we were concerned this would lead to more overdose or deaths” (D&A Service Provider).

### Organisational challenges

Participants noted staff shortages as a particular challenge as demand was surpassing resources available at the time. Participants described the difficulties in recruiting new team members and the pressures of redeploying and redirecting staff to other areas or services that had been particularly impacted by COVID-19.

“That the main issue was, I guess, the difference between delivering kind of supportive services via phone line and by phones than face to face, you know and very quickly, we started srecognising the need to re-train our staff.” (D&A Services Provision Manager).

The pandemic also disrupted communication within and between other organisations as some participants struggled to contact other professionals on online platforms. One participant explained that communicating with pharmacies became challenging as pharmacies were overloaded and exceptionally busy during the pandemic. This became a concern as information on safely reducing supervision (as described above) and legalities needed to be provided to pharmacies. However, there were many barriers to communication, such as changes in contact information as many physical facilities were not up to the expectations of users and services moved to new temporary sites.

### Preserving the quality of care

Participants spoke of different ways through which they had adapted service delivery. While some adaptations were deemed positive (e.g. greater engagement with established service users), others reported challenges in ensuring the quality of care. For example, many adolescent service providers found it difficult to assess over remote consultation methods. They also described that young people had more difficulty engaging with services from their houses as the phone line was not always private.

“We sort of half-expected that young people would want to engage like this, kind of video call, and that would help to pick up on some of that stuff. Nevertheless, actually, what we found, […] was that young people didn’t want that. They wanted phone interventions” (Youth D&A Misuse Service Manager).

Most participants spoke of prioritising risk assessments, emphasising their importance in preserving service user safety.

### Self-reported trend in substance use during the pandemic

Most participants described noticing increases in depressant and sedative use, with Xanax (alprazolam) use increasing amongst adolescent service users, while an increase in alcohol consumption was most prominent amongst adults.

“Cannabis stinks the home. If parents are not down with the weed, […] then they’re gonna get found out, you know, so why not take a couple of Xanax (alprazolam) instead.” (Youth D&A misuse service employee).

It was postulated that the popularity of sedatives rose due to boredom and inaccessibility to mental health services during the lockdown.

“Being isolated, and their way of coping with boredom was drinking alcohol and also mental health-wise, people were sort of like er medicating their mental health with, mainly alcohol” (D&A Misuse Service Regional Manager).

Opiate use was deemed to have reduced, attributed to supply issues and increasing street prices.

“The amounts of […] illicit drugs on the streets actually decreased. And I think the prices went up as well because it’s a supply and demand market.” (D&A Misuse Service Regional Manager).

### Learnings from the pandemic

All participants recognised the benefits of risk-assessing service users using a ‘traffic light system’ and providing care based on each individual’s risk. This is a procedure that participants deemed was likely to continue post-pandemic.

“Think that we've got a body of experience of making those risk assessments and it's going to be informed by things that we learned during the pandemic” (D&A Services Provision Manager)

Most participants agreed that telephone and online interventions may be continued for more stable individuals alongside face-to-face as a ‘blended approach’ as it worked well for both service users and providers in terms of efficiency and ease of accessibility.

Similarly, with individuals on OST who had been trusted with a larger supply of medication to take unsupervised, participants explained this was a positive to come from the pandemic as many enjoyed the responsibility and trust granted to them.

“Some people kind of I think thrived in the pandemic situation you know, actually, you know, took ownership of some of the stuff that they weren't perhaps taking as much control of beforehand and showed us that they were trustworthy, that they were reliable” (D&A Services Provision Manager)

Despite barriers to communication within organisations, several advantages of virtual communication were described, particularly for larger organisations, as it made organising governance and senior management meetings easier.

Participants suggested the importance of having contingency plans in the event of future pandemics. Ensuring staff were correctly trained for such events and developing contingency plans outlining arrangements with various service providers, including community pharmacies, would provide a structured approach. However, most participants noted that in the face of the unprecedented events that followed the pandemic, services managed to continue and adapt predominantly guided by local arrangements and agreements in place.

## Discussion

### Statement of key findings

This study provides insight into the impact of the COVID-19 pandemic on UK-based D&A services. The findings indicate that D&A services had to quickly adapt and restructure all face-to-face interventions as physical premises were closed, operated reduced hours, and introduced social distancing measures. Services adopted a ‘traffic light system’ in order to prioritise and assess risk of each service user. This proved beneficial from the perspective of service providers when reviewing and adjusting treatment regimes. Adaptations were made to prescribing practices and supervised consumption regimes to allow eligible individuals to receive unsupervised take-home doses of OST. However, this presented new challenges such as the risk of overdose or compliance to OST [16]. It was reported, however, that these individuals were more successful with managing their self-reductions at home, suggesting more responsibility can be placed on stable service users for positive outcomes.

### Interpretation

Participants of this study described how most service users responded positively to a reduction in supervised treatments. Improved adherence in the absence of supervision is likely to be psychologically informed, with service users feeling trusted, enhancing their self-esteem. In addition, with less social exposure and hence the perceived stigma, service users likely did not experience stigma-associated demoralisation [18, 19] and exhibited positive SUD-related behaviours.

Mixed experience was observed regarding the provision of remote communication and telehealth. It promoted interprofessional working. However, there were barriers to conducting physical health screenings (e.g., urine-based drug testing), building relationships with new clients, or identifying non-verbal cues.

Participants discussed how a lack of available resources during the initial phases of the COVID-19 pandemic compromised the safety of D&A service providers and users. Lack of sufficient PPE, coupled with increased workload due to staff shortages, heightened stress and anxiety, can result in burnout [20]. Consequently, medication errors also increase, deleteriously affecting patient safety [21]. Furthermore, staff shortages were attributed to the disruption of peripheral D&A services, such as the treatment and screening of hepatitis.

### Further research

The results demonstrate how new practices such as the ‘traffic light system’, unsupervised consumption and online support can be incorporated into the regular care provision. Opportunities exist to further expand the provision of D&A services in community pharmacies beyond the supply of opioid substitution therapy and needle exchange, given their emerging roles as prescribers and established roles around the supply of prescribed and over-the-counter medicines [22–24]. Future research could include a longitudinal study investigating the implications of reduced supervision of people with SUDs in a post-pandemic environment. Moreover, an assessment of the long-term efficacy of digital/telehealth in providing D&A services should be conducted, owing to the divergence of opinions towards its use by participants within this study. In the short term, a comprehensive investigation into the causes of death of people with SUDs during the COVID-19 pandemic should be conducted, as available data are unreported to date, making it difficult to contextualise our findings. This may help inform D&A service providers where resources could be deployed to improve efficiency. In addition, a specific focus on learning the impact of the pandemic on specialist populations such as those experiencing homelessness [25] and patients with dual diagnosis of SUD and mental health problems [26] is needed. It is important to evaluate user experiences and outcomes in those service users who were offered buprenorphine instead of methadone treatment due to practical issues associated with the pandemic such as the lack of urine testing capacity in pharmacy as referred to by some study participants in this study.

### Strengths and Weaknesses

Although a large participant sample was recruited for this qualitative study representing various organisations, there was a lack of adequate response from community pharmacies and rehabilitation centers. This was compounded by most participants belonging to one focus group (N = 35), increasing the propensity of the bandwagon effect and further reducing narrative heterogeneity [27]. Given the large number of participants than anticipated in one focus group, it is likely that every participant was not able to contribute to all topics under discussion. The topic guide that was based on the literature informed data generation. Additionally, responses were based on the entire pandemic spanning several months.

## Conclusion

This study demonstrated the multifaceted impact of the COVID-19 pandemic on UK-based D&A service provisions. Participants described those remote communications improved professional efficiency. However, missed opportunities for screening of blood-borne viruses were noted, and difficulty building relationships with new service users. Participants who provided opioid substitution therapy spoke of service provider/user trust improving following the suspension of daily supervised consumption of opioid substitution therapy. Equally, however, service providers feared deaths due to overdoses. The long-term impact of reduced supervision on SUD treatment and outcomes and any effect of virtual communications on professional efficacy, patient-provider relationships and treatment retention and successes are unknown, suggesting further study to assess their utility.

## Supplementary Information

Below is the link to the electronic supplementary material.

Supplementary file1 (DOCX 19 KB)
