# Supplementary material for: Provision of drug and alcohol services amidst COVID-19 pandemic: a qualitative evaluation on the experiences of service providers
Source: Int J Clin Pharm. 2023 Mar 27;45(5):1098–106. doi: 10.1007/s11096-023-01557-1 (PMC10042098; doi:10.1007/s11096-023-01557-1)
Supplement: Supplementary file 1 — Supplementary file1 (DOCX 19 KB) [file 11096_2023_1557_MOESM1_ESM.docx]

Electronic supplemental material 1

Topic guide: Provision of drug and alcohol services during COVID-19 pandemic: Perspectives of service providers

**Topic Questions**

1. What were the general experiences of providing drug and alcohol services during the covid-19 pandemic?
2. Based on your own experiences, did you find that lockdown orders affected the delivery of care for patients with substance use disorders, and if so, how? Please elaborate.
   - 1. E.g., Counselling patients on the correct use of medicines, delivery of medicines to patients infected with COVID-19 etc.
     2. What was the procedure if medicines were not collected/could not be collected
3. How were difficulties in the delivery of care overcome or minimised during lockdown(E.g. Changes to way of working, improvisations etc.)?
4. Were there any aspects of the delivery of care for patients which were simplified due to the lockdown orders which were introduced?
   - 1. E.g., Remote communications, telephone appointments etc.
5. **[For participants who worked in residential rehabilitation service provision]** – How was the capacity of rehabilitation facilities affected during the pandemic due to social distancing and how was this handled regarding the current occupants?
6. In your own opinion, do you believe that the quality of care offered to patients with substance use disorders changed during the pandemic? Please elaborate.
7. During lockdown, did you observe any changes to the rates of poisonings from people substituting one substance for another, and were there any changes to how these patients were cared for?
   - 1. E.g., Ingestion of residual methanol from home-brewed alcohol, abuse of chemicals not intended for human consumption?
8. How was communication and working between teams treating substance use disorders and other relevant professional agencies (e.g., Other healthcare teams, social care teams, police etc.) affected by the pandemic? Please elaborate.
9. During the pandemic, were there any changes in the attitudes of staff towards patients or vice versa? Please elaborate.
10. Compared to before the pandemic, how would you describe the current numbers of people seeking help for substance use disorders? Please elaborate.
11. Do you believe that current services are equipped to sufficiently care for all patients who currently have substance misuse disorders and who may develop them because of the socioeconomic changes arising from the pandemic? Please elaborate.
12. With the benefit of hindsight, what measures could have been introduced prior to the outbreak of COVID-19 pandemic which would have minimised disruption to the delivery of care of patients with substance use disorders?
13. Are there any new practises which developed through the course of the pandemic which will remain in place into the future?
14. Do you have anything more to add?

Thank you for your time and participation- it is greatly appreciated.
